# Analog vs. digital: geschlechterspezifische Wahrnehmung von Simulationstraining in der endovaskulären Ausbildung

**DOI:** 10.1055/a-2651-3266

**Published:** 2025-07-17

**Authors:** David Wippel, Miar Ouaret, Maximilian Lutz, Laura Schönherr, Kristina Grassl, Juliana Heidler, Inez Ohashi Torres, Elke R. Gizewski, Florian K. Enzmann, Sabine Wipper

**Affiliations:** 1 27280 Universitätsklinik für Gefäßchirurgie, Medizinische Universität Innsbruck, Innsbruck, Österreich; 2 27280 Universitätsklinik für Radiologie, Medizinische Universität Innsbruck, Innsbruck, Österreich; 3 Gefäßchirurgie, Universitätsklinikum der Medizinischen Fakultät der Universität Sao Paulo, Ribeirao Preto, Brasilien

**Keywords:** Simulation, Simulatortraining, endovaskuläres Training, 3D-gedruckt, simulation, simulation based training, endovascular training, 3D printed

## Abstract

**Hintergrund:**

Trotz eines hohen Anteils weiblicher Absolvent*innen in der Humanmedizin sind Frauen in der Chirurgie nach wie vor auf höheren Karrierestufen unterrepräsentiert. Studien zeigen geschlechtsspezifische Unterschiede in der operativen Autonomie, Selbsteinschätzung und psychischen Belastung. Simulationstraining könnte einen Beitrag zur Reduktion dieser Disparitäten leisten. Ziel dieser Studie war es, geschlechterspezifische Unterschiede in der Bewertung und Wahrnehmung verschiedener endovaskulärer Trainingsmodelle zu untersuchen.

**Material und Methoden:**

Zwischen März und August 2024 wurden am Universitätsklinikum Innsbruck 32 Ärzt*innen (16 weiblich, 16 männlich) aus der Gefäßchirurgie und Radiologie in eine prospektive Studie eingeschlossen. Alle Teilnehmenden führten eine standardisierte Beckenarterien-Intervention an 3 Modellen durch: einem flexiblen und einem steifen 3D-gedruckten Gefäßmodell sowie einem digitalen Simulator. Die Bewertung erfolgte mittels Fragebogen zu Augenscheinvalidität, Konstruktvalidität und konkurrierender Validität. Es wurden geschlechtsspezifische Unterschiede statistisch analysiert.

**Ergebnisse:**

Es zeigten sich keine geschlechtsspezifischen Unterschiede in der Bewertung der Augenscheinvalidität oder der Modellpräferenzen. Bei der Konstruktvalidität hingegen bewerteten weibliche Teilnehmende den Nutzen der Trainingsmodelle signifikant höher (p < 0,05). Zudem wiesen Männer eine höhere praktische Interventionserfahrung auf, obwohl keine Unterschiede in der Berufserfahrung bestanden. Beide Geschlechter sprachen sich überwiegend für ein regelmäßiges Simulationstraining aus.

**Schlussfolgerung:**

Simulationstraining wird geschlechtsübergreifend als zentrales Lerninstrument anerkannt. Die höhere Bewertung der Konstruktvalidität durch Frauen deutet auf dessen Bedeutung als kompensatorisches Werkzeug zur Förderung operativer Kompetenzen hin. Eine strukturierte Integration von Simulationstraining könnte dazu beitragen, bestehende Trainingsdisparitäten in der endovaskulären Weiterbildung zu reduzieren.

## Abstract (en)

**Purpose:**

Despite the increasing number of female medical graduates, women remain underrepresented in surgical leadership roles. Previous studies have highlighted gender disparities in operative autonomy, self-assessment, and psychological burden. Simulation-based training may offer a means to reduce such disparities. This study aimed to assess gender-specific differences in the evaluation of various endovascular training models.

**Materials and Methods:**

Between March and August 2024, 32 physicians (16 female, 16 male) from vascular surgery and radiology departments participated in a prospective study at the University Hospital in Innsbruck. Each participant performed a standardised iliac artery procedure using three training models: a flexible and a stiff 3D-printed vascular model, and a digital simulator. A structured questionnaire evaluated face validity, construct validity, and concurrent validity. Gender differences were analysed statistically.

**Results:**

There were no significant gender differences in face validity ratings or model preferences. However, female participants consistently rated the construct validity of all models significantly higher (p < 0.05). Male participants had greater procedural experience, despite similar durations of professional experience. Both genders expressed strong support for regular simulation-based training.

**Conclusion:**

Simulation-based training is broadly recognised as a key educational tool across genders. The higher construct validity ratings by female participants suggest that simulation is perceived as a compensatory method for developing procedural skills. Structured implementation of simulation training may help reduce disparities in endovascular education.

## Einleitung

Seit 2004 stellen Frauen über die Hälfte der Medizinabsolvent*innen in Deutschland, was eine bedeutende Verschiebung in der Geschlechterverteilung des medizinischen Nachwuchses darstellt. Dieser Trend setzt sich zunächst in der chirurgischen Weiterbildung fort, wo laut Angaben der Bundesärztekammer von 2021 der Frauenanteil bei etwa 58% liegt. Im weiteren Karriereverlauf reduziert sich dieser jedoch drastisch: Nur noch rund 26% der Oberarztpositionen in chirurgischen Fachdisziplinen sind weiblich besetzt, auf Leitungsebene sinkt dieser Anteil auf etwa 10%. Eine ähnliche Entwicklung zeigt sich in der Gefäßchirurgie: 2021 waren lediglich 26,5% der Fachärzt*innen Frauen, von denen nur 9% eine Leitungsposition innehatten. Diese Diskrepanz zwischen anfänglichem Frauenanteil und späterer Karriereentwicklung könnte auf tief verwurzelte strukturelle Hürden hindeuten, die den beruflichen Aufstieg von Chirurginnen erschweren 1 .

Studien zeigen, dass Frauen in der chirurgischen Weiterbildung höhere Abbruchraten als ihre männlichen Kollegen aufweisen und häufiger unter dem Phänomen des „Stereotype Threat“ leiden – die Angst, negative geschlechtsspezifische Stereotype zu bestätigen. Dies geht mit erhöhtem psychischem Druck und einer höheren Belastung einher, was wiederum mit einer gesteigerten Anfälligkeit für Burn-out und suizidale Gedanken assoziiert ist 2 3 4 .

Hinzu kommt, dass Chirurginnen ihre eigene Kompetenz in der Selbsteinschätzung tendenziell schlechter bewerten als ihre männlichen Kollegen und häufiger unter dem Impostor-Syndrom leiden – einer kognitiven Verzerrung, die durch Selbstzweifel und die unbegründete Angst vor Entlarvung als inkompetent geprägt ist 5 6 7 8 .

Auch zeigt sich in der Literatur, dass Frauen trotz vergleichbarer chirurgischer Leistungsbewertungen im Verlauf ihrer Weiterbildung zunehmend weniger operative Autonomie erhalten 9 10 11 .

Diese Faktoren haben nicht nur individuelle Konsequenzen, sondern beeinflussen auch die Geschlechterverteilung in chirurgischen Fachdisziplinen insgesamt. Ein vielversprechender Lösungsansatz könnte im Simulationstraining liegen. Während erste Studien geschlechterspezifische Unterschiede in den visuospatialen Fähigkeiten zu Beginn des Trainings zeigen, gleichen gezielte Simulatortrainings diese Unterschiede aus 12 13 .

Trotz der hohen Akzeptanz solcher Trainingskonzepte in deutschen Kliniken – 94,5% der befragten Häuser befürworten deren Einsatz – und der zunehmenden Berücksichtigung der Empfehlung zur Integration von Simulatortraining in die Ausbildung, wie in den aktuellen europäischen Aortenguidelines ersichtlich, fehlt bislang eine systematische Implementierung in die chirurgische Weiterbildung 14 15 .

Eine solche könnte jedoch dazu beitragen, gleiche Lernchancen für alle Assistenzärzt*innen zu schaffen und bestehende Diskrepanzen in der Ausbildung auszugleichen. Ziel dieser Studie ist daher die Evaluierung geschlechterspezifischer Unterschiede in der Wahrnehmung und Nutzung verschiedener Trainingsmodelle in der endovaskulären Ausbildung – sowohl traditioneller digitaler Simulatoren als auch neuartiger 3D-gedruckter Modelle. Die gewonnenen Erkenntnisse sollen zur Entwicklung gerechter und gleichwertiger Ausbildungsstrategien beitragen.

## Methodik

### Studiendesign

**Abb. 1:**
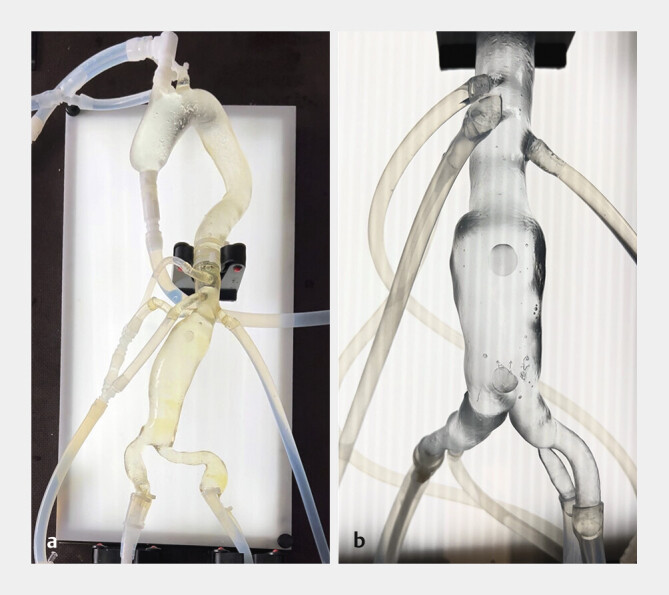
Versuchsaufbau der 3D-gedruckten Aortenmodelle. **a** Flexibles Stereolithografie-Modell aus 80A-Shore-Harz. **b** Steifes Stereolithografie-Modell aus Clear V5-Harz. Beide Modelle sind an eine pulsatile Flüssigkeitspumpe angeschlossen, um den physiologischen Blutfluss zu simulieren, und auf einer Interventionseinheit mit Hintergrundbeleuchtung fixiert.

Die Studie wurde zwischen März und August 2024 am Universitätsklinikum Innsbruck durchgeführt. Dabei wurden 3 endovaskuläre Trainingsmodelle untersucht: ein flexibles und ein steifes 3D-gedrucktes Kunstharzmodell sowie ein digitaler Simulator. Die beiden 3D-gedruckten Modelle stellten die thorakoabdominelle Aorta sowie die Iliakalgefäße dar und wurden mithilfe eines Stereolithografie-Druckverfahrens aus segmentierten Computertomografiedaten mit einem 3D-Drucker der Firma Formlabs (Formlabs GmbH, Berlin, Deutschland) gefertigt. Als Druckmaterialien kamen das transparente, elastische 80A Resin sowie das transparente, steife Clear V5 Resin zum Einsatz. Anschließend wurden die Modelle an eine pulsatile Flüssigkeitspumpe angeschlossen, um den physiologischen Blutfluss zu simulieren, und auf einer Interventionseinheit mit Hintergrundbeleuchtung fixiert, die optimale Sichtverhältnisse während der Eingriffe gewährleistete. Abb. 1 zeigt den Versuchsaufbau der beiden 3D-gedruckten Modelle. Als digitaler Referenzstandard diente der etablierte Mentice-Endovaskularsimulator (Mentice AB, Göteborg, Schweden).

Eingeschlossen wurden Ärzt*innen der Abteilungen für Gefäßchirurgie sowie Radiologie mit unterschiedlichen Erfahrungsstufen in endovaskulären Interventionen, die alle zuvor bei realen endovaskulären Eingriffen assistiert oder diese selbst durchgeführt hatten. Es wurden keine zusätzlichen Ausschlusskriterien angewendet. Jeder Teilnehmende führte Interventionen an allen 3 Modellen durch, wobei die Reihenfolge randomisiert wurde, um eine ausgewogene Exposition sicherzustellen.

Die Teilnehmenden führten in jedem Modell eine *Cross-over* -Ballonangioplastie der A. iliaca externa durch, wobei zunächst die kontralaterale A. iliaca interna sondiert wurde. Diese Intervention wurde gewählt, da sie zentrale endovaskuläre Fähigkeiten für aortale und periphere Eingriffe abbildet. Sie umfasst die Nutzung verschiedener Katheter- und Schleusensysteme, die Navigation durch große (A. iliaca communis sowie Aorta) und kleine Gefäße (A. iliaca interna) sowie den Einsatz interventioneller Instrumente, wie eines PTA-Ballons.

Für die 3D-gedruckten Modelle standen gängige interventionelle Instrumente, einschl. Katheter, Führungsdrähte und Schleusen, physisch zur Verfügung. Im digitalen Simulator erfolgte die Materialauswahl hingegen virtuell, wobei ein realer Katheter und Führungsdraht stellvertretend für das gewählte digitale Equipment dienten.

### Fragebogen und Datenerhebung

Nach Abschluss der Prozeduren bewerteten die Teilnehmenden die Modelle mithilfe eines standardisierten Fragebogens, der über REDCap (Vanderbilt University, TN, USA) bereitgestellt wurde. Der Fragebogen fokussierte auf 3 zentrale Validitätskategorien:

- Augenscheinvalidität: Beurteilung der anatomischen Präzision des Gefäßzugangs, des Widerstandsgefühls, des haptischen Feedbacks sowie der allgemeinen Realitätsnähe der Modelle auf einer Likert-Skala von 1 bis 10.
- Konstruktvalidität: Bewertung des Potenzials der Modelle zur Förderung von Fähigkeiten, der Wahrscheinlichkeit ihrer Wiederverwendung, ihres Nutzens und Sinnhaftigkeit für die Ausbildung und der allgemeinen Zufriedenheit, ebenfalls auf einer Likert-Skala von 1 bis 10.
- Konkurrierende Validität: Identifikation des Modells, das als realistischstes, nützlichstes und am wahrscheinlichsten wiederverwendbares Modell wahrgenommen wurde, basierend auf einem Single-Choice-Format.

Das primäre Ziel der Studie bestand darin, Unterschiede in den Bewertungen der 3 Validitätskategorien zwischen den Modellen zu analysieren. Zusätzlich wurden geschlechtsspezifische Präferenzen sowie die Variabilität der Antworten in Abhängigkeit vom Geschlecht untersucht.

Diese Studie erweitert bestehende Arbeiten dieser Forschungsgruppe, indem sie eine geschlechtsspezifische Perspektive integriert und untersucht, wie männliche und weibliche Teilnehmende endovaskuläre Trainingsmodelle unterschiedlich bewerten.

### Statistische Analyse

Zur Untersuchung geschlechtsspezifischer Unterschiede in den Bewertungen der Augenscheinvalidität, Konstruktvalidität und konkurrierenden Validität zwischen den 3 Simulationsmodellen wurden statistische Analysen durchgeführt. Alle Berechnungen erfolgten mit Python (Version 3.11), wobei ein Signifikanzniveau von p < 0,05 festgelegt wurde.

Da die Bewertungen für Augenscheinvalidität und Konstruktvalidität auf ordinalen Skalen erhoben wurden, kamen nichtparametrische Testverfahren zum Einsatz. Zur Analyse von Unterschieden zwischen den 3 Modellen innerhalb der jeweiligen Geschlechtsgruppen wurde der Kruskal-Wallis-Test verwendet. Paarweise Post-hoc-Vergleiche wurden mittels Dunn-Test mit Bonferroni-Korrektur durchgeführt, um spezifische Gruppenunterschiede zu identifizieren.

Um geschlechtsspezifische Unterschiede in den Validitätsbewertungen zu ermitteln, wurde der Mann-Whitney-U-Test eingesetzt, der die Verteilung der Werte für jedes Modell und jede Validitätskategorie zwischen männlichen und weiblichen Teilnehmenden vergleicht. Derselbe Test wurde auch zur Analyse von ordinalen Daten wie der bevorzugten Trainingsfrequenz verwendet. Die Effektgrößen wurden mithilfe der biserialen Rangkorrelation berechnet, um die Stärke der beobachteten Unterschiede zu quantifizieren.

Zur Überprüfung der Varianzhomogenität zwischen den Geschlechtern hinsichtlich der Augenscheinvaliditäts- und Konstruktvaliditätsbewertungen wurde der Levene-Test durchgeführt. Die Ergebnisse wurden ergänzend durch eine visuelle Analyse mithilfe von Boxplots veranschaulicht.

Bei den Fragen zur konkurrierenden Validität, die kategoriale Daten umfassten und eine Auswahl eines bevorzugten Modells erforderten, wurde der Chi-Quadrat-Unabhängigkeitstest angewendet, um geschlechtsspezifische Unterschiede zu untersuchen. Hierzu wurden Kontingenztafeln für die Vergleichsparameter Realismus, Benutzerfreundlichkeit, Wiederverwendungswahrscheinlichkeit sowie die Präferenz für regelmäßiges Training erstellt.

## Resultate

**Abb. 2:**
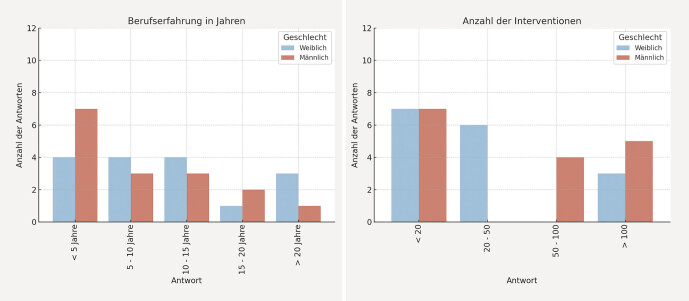
Geschlechtsspezifische Verteilung der beruflichen Erfahrung und der Anzahl durchgeführter Interventionen.

Alle 32 Teilnehmenden (16 Frauen, 16 Männer) schlossen die Studie erfolgreich ab. Die Verteilung der beruflichen Erfahrung und der durchgeführten Interventionen ist in Abb. 2 dargestellt.

Zwischen den Geschlechtern ergaben sich keine signifikanten Unterschiede in den absolvierten Berufsjahren (p = 0,656, Mann-Whitney-U-Test). Allerdings zeigte sich eine signifikante Differenz in der Interventionserfahrung (p = 0,015): Männliche Teilnehmende waren häufiger in den Kategorien mit über 50 durchgeführten Eingriffen vertreten, während sich weibliche Teilnehmende überwiegend in der Kategorie bis zu 50 Eingriffen befanden.

**Abb. 3:**
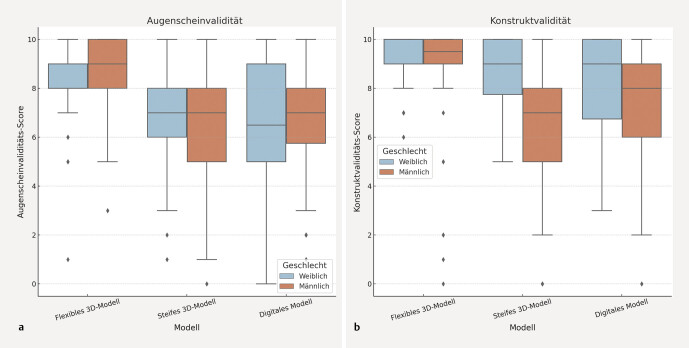
Boxplot der geschlechtsspezifischen Verteilung der kombinierten Likert-Skalen-Werte für ( **a** ) Augenscheinvalidität und ( **b** ) Konstruktvalidität über alle Simulationsmodelle hinweg.

Die kombinierten Bewertungen der Augenschein- und Konstruktvalidität für beide Geschlechtergruppen sind in Abb. 3 visualisiert.

Der Mann-Whitney-U-Test ergab keine signifikanten geschlechtsspezifischen Unterschiede in den Bewertungen der Augenscheinvalidität für das flexible 3D-Modell (p = 0,069), das steife 3D-Modell (p = 0,747) oder den digitalen Simulator (p = 0,957). Im Gegensatz dazu zeigten sich signifikante Geschlechtsunterschiede in den Bewertungen der Konstruktvalidität über alle 3 Modelle hinweg: flexibles 3D-Modell (p = 0,039), steifes 3D-Modell (p < 0,001) und digitaler Simulator (p = 0,01), wobei weibliche Teilnehmende durchgehend höhere Medianwerte vergaben.

Vergleichende Varianzanalysen mithilfe des Levene-Tests bestätigten diese Unterschiede. Für die Augenscheinvalidität zeigte sich eine signifikante Variabilität nur im flexiblen 3D-Modell (p = 0,05), während für das steife 3D-Modell und den digitalen Simulator keine signifikanten Unterschiede in der Streuung beobachtet wurden. Im Bereich der Konstruktvalidität waren die Unterschiede in der Variabilität für alle 3 Modelle signifikant, zurückzuführen auf die höheren Bewertungen der selbigen durch die weiblichen Teilnehmenden.

Hinsichtlich der konkurrierenden Validität ergaben sich keine signifikanten geschlechtsspezifischen Unterschiede in den Bewertungen der Realitätsnähe (p = 0,143), der Benutzerfreundlichkeit (p = 0,095) oder der Wiederverwendungswahrscheinlichkeit (p = 0,71) der 3 Modelle. Gut ⅔ der Teilnehmer empfanden die 3D-gedruckten Modelle als realitätsnäher als das digitale Gegenstück, bez. der Benutzerfreundlichkeit und Wiederverwendungswahrscheinlichkeit bevorzugten in etwa die Hälfte der Proband*innen die 3D-gedruckten Modelle und die andere Hälfte beurteilte die beiden Modalitäten als ebenbürtig. Ebenso wurden keine signifikanten Differenzen hinsichtlich des Wunsches nach regelmäßigem Training (p = 0,562) oder der bevorzugten Trainingsfrequenz (p = 0,726) festgestellt. Unabhängig vom Geschlecht sprachen sich 86% der Teilnehmenden für ein regelmäßiges Training aus, das typischerweise einmal pro Quartal stattfinden sollte.

## Diskussion

Die Ergebnisse dieser Studie zeigen, dass sich männliche und weibliche Teilnehmende in ihrer Bewertung endovaskulärer Trainingsmodelle hinsichtlich der Augenscheinvalidität und konkurrierenden Validität weitgehend einig waren, jedoch signifikante geschlechtsspezifische Unterschiede in der Konstruktvalidität auftraten. Weibliche Teilnehmende bewerteten den Nutzen der Modelle für die Fähigkeitsentwicklung durchgängig höher als ihre männlichen Kollegen. Dies deutet darauf hin, dass Frauen Simulationstraining möglicherweise als essenzielles Instrument zur Kompensation bestehender struktureller Nachteile sowie der geringeren Exposition in der klinischen Ausbildung wahrnehmen.

Die Analyse der konkurrierenden Validität ergab keine signifikanten geschlechtsspezifischen Unterschiede in der Bewertung der Modelle. Männer und Frauen bewerteten sowohl die digitalen als auch die 3D-gedruckten Simulatoren ähnlich in Bezug auf Realismus, Benutzerfreundlichkeit und Wiederverwendung, was darauf hindeutet, dass beide Simulatormodalitäten geschlechtsübergreifend als geeignete Lernwerkzeuge wahrgenommen wurden, mit einer Präferenz hin zu den 3D-gedruckten Modellen.

Darüber hinaus bestätigte sich in dieser Studie eine geschlechtsspezifische Ungleichverteilung der praktischen Erfahrung: Während männliche Teilnehmende überproportional häufig in den Kategorien mit mehr als 50 durchgeführten Interventionen vertreten waren, fanden sich weibliche Teilnehmende überwiegend im Bereich von ≤ 50 Eingriffen wieder, was eine signifikante Diskrepanz in der praktischen Exposition nahelegt. Interessanterweise bestanden keine Unterschiede in der Berufserfahrung zwischen den Geschlechtern, was darauf hindeutet, dass nicht allein die Anzahl der Ausbildungsjahre, sondern vielmehr strukturelle Faktoren wie ungleiche Zuweisung praktischer Lernmöglichkeiten eine Rolle spielen könnten.

Diese Befunde stehen im Einklang mit früheren Studien, die zeigen, dass Assistenzärztinnen in chirurgischen Disziplinen tendenziell weniger operative Autonomie erhalten als ihre männlichen Kollegen, obwohl ihre Leistungsbewertungen keine signifikanten Unterschiede aufweisen 9 10 11 . Dies deutet darauf hin, dass wahrgenommene Defizite in der chirurgischen Selbstständigkeit nicht mit objektiven Leistungskriterien korrelieren, sondern vielmehr durch ungleiche Ausbildungsstrukturen bedingt sind.

Ein zusätzlicher Faktor ist der Mangel an weiblichen Vorbildern und Mentorinnen, der die Karriereentwicklung von Nachwuchschirurginnen hemmt. Mentoring gilt als essenzieller Karrierebooster, da es operative Fertigkeiten stärkt, Karrierewege strukturiert und den Zugang zu Netzwerken erleichtert. Fehlen weibliche Mentorinnen, fehlt oft auch die gezielte Förderung, die Frauen auf ihrem Weg in Führungspositionen unterstützt. Eine verstärkte Implementierung von Mentoring-Programmen könnte daher entscheidend zur Chancengleichheit in der chirurgischen Karriere beitragen 1 16 17 18 .

Die durchweg höheren Bewertungen der Konstruktvalidität durch weibliche Teilnehmende könnten darauf hindeuten, dass sie Simulationstraining als entscheidendes Mittel zur Angleichung operativer Erfahrung und zur Reduktion bestehender Trainingsdefizite wahrnehmen. Dies deckt sich mit der Literatur, die zeigt, dass Simulatortraining insbesondere für unterrepräsentierte Gruppen eine wertvolle Ergänzung zur klassischen operativen Ausbildung darstellt und es ermöglicht, evtl. bestehende Unterschiede im Trainingsstand auszugleichen und langfristig gleichwertige Kompetenzen zu fördern 12 13 .

Obwohl männliche und weibliche Teilnehmende sich gleichermaßen für regelmäßige Simulationstrainings aussprachen, könnte die höhere Bedeutung, die Frauen dem Simulationstraining beimessen, auf eine ungleiche Verteilung realer Eingriffsmöglichkeiten hinweisen. Männer, die traditionell weniger Barrieren im Zugang zu operativer Erfahrung erleben, könnten Simulationstraining als weniger kritisch für ihre Kompetenzentwicklung ansehen.

Um bestehende Disparitäten zu reduzieren, sollten verstärkt strukturierte Simulationstrainings implementiert werden, die gezielt darauf abzielen, unterrepräsentierte Gruppen – insbesondere Frauen – operativ zu stärken. Darüber hinaus sind Maßnahmen erforderlich, die die operative Autonomie von Chirurginnen im klinischen Alltag fördern, darunter transparente Zuteilungskriterien für Eingriffe, Mentorship-Programme und gezielte Förderung von Frauen in chirurgischen Führungspositionen.

## Limitationen

Eine wesentliche Limitation dieser Studie ist die relativ geringe Stichprobengröße, die möglicherweise die Generalisierbarkeit der Ergebnisse einschränkt. Zukünftige Forschung sollte mit größeren Kohorten erfolgen, um geschlechtsspezifische Unterschiede in der chirurgischen Ausbildung noch detaillierter zu analysieren. Darüber hinaus wäre eine Langzeituntersuchung sinnvoll, um den Einfluss von Simulationstraining auf die tatsächliche operative Kompetenzentwicklung und Karriereverläufe von Assistenzärzt*innen zu untersuchen.

## Schlussfolgerung

Simulationstraining wird geschlechtsübergreifend als essenzielles Lerninstrument anerkannt, und der Wunsch nach regelmäßigen Trainingseinheiten besteht. Es zeigten sich keine geschlechtsspezifischen Unterschiede in der Präferenz für digitale oder 3D-gedruckte Simulatoren. Unabhängig vom Trainingsmodell bewerteten Frauen dessen Nutzen für die Fähigkeitsentwicklung durchgängig höher. Die Ergebnisse unterstreichen die Notwendigkeit einer systematischen Integration von Simulationstraining in die chirurgische Weiterbildung, um eine gleichberechtigte praktische Ausbildung zu fördern und bestehende Unterschiede in der operativen Exposition auszugleichen.
